# Acupuncture Stimulation at GB34 Restores MPTP-Induced Neurogenesis Impairment in the Subventricular Zone of Mice

**DOI:** 10.1155/2017/3971675

**Published:** 2017-05-16

**Authors:** Hyongjun Jeon, Sun Ryu, Dongsoo Kim, Sungtae Koo, Ki-Tae Ha, Seungtae Kim

**Affiliations:** ^1^Department of Korean Medical Science, School of Korean Medicine, Pusan National University, Yangsan, Gyeongsangnam-do 50612, Republic of Korea; ^2^Korean Medicine Research Center for Healthy Aging, Pusan National University, Yangsan, Gyeongsangnam-do 50612, Republic of Korea

## Abstract

Adult neurogenesis has recently been considered a new therapeutic paradigm of Parkinson's disease. In this study, we investigated whether acupuncture restores 1-methyl-4-phenyl-1,2,3,6-tetrahydropyridine- (MPTP-) induced impaired neurogenesis in the subventricular zone (SVZ). Male C57BL/6 mice were given 30 mg/kg of MPTP intraperitoneally once a day for 5 days, after which they were intraperitoneally injected with 50 mg/kg of bromodeoxyuridine (BrdU) and given acupuncture stimulation at HT7 or GB34 for 12 consecutive days. Dopaminergic neuronal survival in the nigrostriatal pathway and cell proliferation in the SVZ was then evaluated by immunostaining. MPTP administration induced dopaminergic neuronal death in the nigrostriatal pathway, which was suppressed by acupuncture stimulation at GB34. MPTP administration also suppressed the number of BrdU-positive cells and glial fibrillary acidic protein/BrdU-positive cells and increased the number of doublecortin/BrdU-positive cells in the SVZ, which were restored by acupuncture stimulation at GB34. These results indicate that acupuncture stimulation at GB34 restores MPTP-induced neurogenesis impairment.

## 1. Introduction

Parkinson's disease (PD) is a frequently observed neurodegenerative disease that afflicts the elderly and is characterized by selective loss of dopaminergic (DA) neurons of substantia nigra (SN) and striatum [1, 2]. DA neurons play a central role in the control of motor functions and their loss causes behavioral dysfunctions such as akinesia, tremor, bradykinesia, rigidity, and postural instability [3]. It is well known that several factors including aging, inflammation, and oxidative stress can trigger PD, but the mechanism remains unclear [4].

Neurogenesis, which is the process by which neurons are generated from neural stem cells and progenitor cells, has been shown to occur in special zones of adult brain including the subventricular zone (SVZ) of the lateral ventricles and the dentate gyrus of the hippocampus [5–7]. Interestingly, reduced proliferation of SVZ cells was observed in PD patients [8], suggesting that PD suppresses neurogenesis in the brain. Promotion of neurogenesis and replacement of dead DA neurons with new ones in the brains of PD patients would help alleviate PD symptoms. Therefore, adult neurogenesis has recently been considered a new therapeutic paradigm of PD.

When neurogenesis occurs, several types of progenitor cells are observed. Type A cells are proliferating neuroblasts that form chains to migrate, while type B cells are similar to astrocytes morphologically [9]. Doublecortin (DCX), a brain-specific microtubule-associated protein [10], is considered a marker of type A migratory neuroblasts, while glial fibrillary acid protein (GFAP) is a marker of type B astrocytic cells.

Acupuncture is widely used in East Asia and considered an alternative therapy for PD [11], although recent studies have shown that acupuncture treatment has neuroprotective effects and alleviates behavioral dysfunctions in a PD mouse model [12, 13]. And recently, acupuncture has been suggested as a possible therapeutic tool for treatment of neurodegenerative diseases by inducing neurogenesis [14]. For example, electroacupuncture enhances neuronal differentiation in rat brains [15] and endogenous neurogenesis in an ischemic stroke animal model [16]. A systematic review concluded that the neuroprotective effect of acupuncture on experimental ischemic stroke is positively correlated with neurogenesis [17]. Based on these results, it is possible that acupuncture can enhance neurogenesis in the brains of PD animal models although there is no direct evidence on PD animal model.

Therefore, in this study, we investigated whether acupuncture treatment can promote neurogenesis in SVZ of 1-methyl-4-phenyl-1,2,3,6-tetrahydropyridine- (MPTP-) treated mice and which types of cells increased as a result of neurogenesis.

## 2. Material and Methods

### 2.1. Animals and Groups

This study was approved by the Pusan National University Institutional Animal Care and Use Committee. Male nine-week-old C57BL/6 mice (Orient Bio Inc., Seongnam, Korea) weighing 20–23 g were housed at room temperature (22 ± 2°C) under a standard 12-hour light/dark cycle with unlimited access to food and water. The animals were handled in accordance with the current guidelines established by the National Institutes of Health (NIH) Guide for the Care and Use of Laboratory Animals (NIH Publication number 85-23, 1985). Mice were randomly assigned to four groups: a saline-injected group (Saline, *n* = 6), a MPTP-injected group (MPTP, *n* = 7), a MPTP-injected plus acupuncture-treated at HT7 group (HT7, *n* = 6), and a MPTP-injected plus acupuncture-treated at GB34 group (GB34, *n* = 7).

### 2.2. MPTP Administration and Acupuncture Stimulation

All mice except the saline group received an intraperitoneal injection of MPTP-HCl (30 mg/kg; Sigma, St. Louis, MO, USA) in sterilized normal saline at 24-hour intervals for 5 consecutive days. The mice in the saline group received intraperitoneal injection of vehicle on the same schedule. Two hours after the MPTP or saline injection, all mice received intraperitoneal injection of 50 mg/kg of bromodeoxyuridine (BrdU; Sigma) once a day for 12 consecutive days at 24 h intervals.

**Figure 1:**
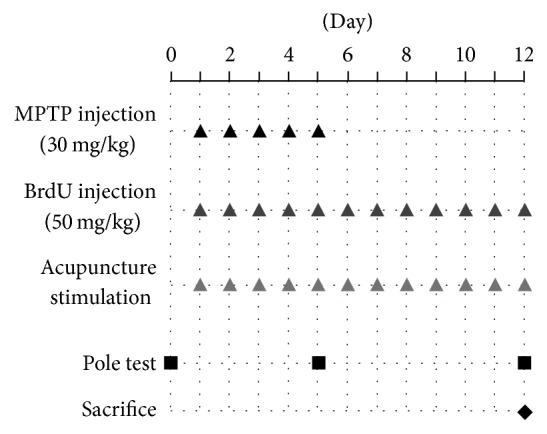
The schedule diagram of each experiment. MPTP or saline was intraperitoneally injected into mice daily for five consecutive days. BrdU injection was performed 2 h after each MPTP or saline injection and continued daily until 7 days after the final MPTP or saline injection. Two hours after the BrdU injection, mice in acupuncture-treated group received acupuncture stimulation at GB34 or SI3. The pole test was performed one day before MPTP injection (day 0), 2 h after the fifth acupuncture stimulation (day 5), and 2 h after the last acupuncture stimulation (day 12).

Two hours after the BrdU injection, mice in the HT7 group received acupuncture stimulation at HT7 and those in the GB34 group received stimulation at GB34. HT7 is located at the end of the transverse crease of the ulnar wrist of the forepaw, and GB34 is located at point of intersection of lines from the anterior borders to the head of the fibula [18]. The mice in the HT7 and GB34 groups were lightly immobilized, and stainless acupuncture needles (0.18 mm × 8 mm; Dongbang acupuncture, Boryeng, Korea) were inserted to a depth of 3 mm at the HT7 (HT7 group) or GB34 (GB34 group) acupoint, turned at a rate of two revolutions per second for 30 s, and removed immediately afterward. Mice underwent this procedure from left to right acupoints in sequence (treatment lasting 60 s). This treatment continued at 24-hour intervals for 12 consecutive days. Mice in the saline and the MPTP groups were also lightly immobilized for 60 s without acupuncture and then returned to their cage (Figure 1).

### 2.3. Behavior Test

The pole test was performed one day before MPTP injection (day 0), 2 h after the fifth acupuncture stimulation (day 5), and 2 h after the last acupuncture stimulation (day 12). Briefly, mice were mounted head-downwards near the top of a rough-surfaced wooden pole (10 mm in diameter and 55 cm in height), and the time taken to reach the bottom of the pole was measured [18]. The test was repeated three times at 30 s time intervals, after which behavioral changes were evaluated according to the average of the three times.

### 2.4. Immunohistochemistry

The mice were sacrificed 2 h after the last pole test on day 12. Mice were anesthetized by isoflurane (JW Pharmaceutical, Seoul, Republic of Korea) and then perfused transcardially with 4% paraformaldehyde dissolved in 0.1 M phosphate buffer (PFA). Next, the brain was quickly harvested, postfixed in 4% PFA for 48 hours, and immersed in 30% sucrose solution for storage at 4°C prior to sectioning. Frozen sections were cut to a thickness of 40 *μ*m using a cryostat (Leica Microsystems, Wetzlar, Germany). The SN sections located between AP −3.08 and −3.28 mm from the bregma and the striatum sections located between AP +0.48 and +0.68 mm from the bregma were incubated with 1% H_2_O_2_ in 0.05 M phosphate-buffered saline for 15 min, followed by 0.3% Triton X-100 and 3% normal blocking serum in PBS at room temperature for 1 h, and then stained overnight at room temperature using an antityrosine hydroxylase (TH, 1 : 200; Santa Cruz Biotechnology, Santa Cruz, CA, USA) primary antibody. The next day, sections were incubated with Vectastain Elite ABC reagents (Vector Laboratories Inc., Burlingame, CA, USA) at room temperature for 1 h and then incubated with a diaminobenzidine substrate kit (Vector Laboratories Inc.) for 5 min. The tissues were subsequently mounted on gelatin-coated slides, air-dried, dehydrated, and coverslipped. Histological pictures were taken by an Axio Scope.A1 microscope (Carl ZEISS, Oberkochen, Germany) and an AxioCam ICc3 camera (Carl ZEISS). The survival of DA neurons was evaluated by the number of TH-positive neuronal cells in SN and by the mean value of the optical density (OD) in ST using Image-Pro Plus 6.0 (Media Cybernetics, Silver Spring, MD, USA).

### 2.5. Immunofluorescent Staining

After blocking, all sections were first blocked with 10% normal serum-blocking solution species the same as the secondary antibody containing 3% (w/v) bovine serum albumin, 0.1% Triton X-100, and 0.05% Tween-20 for 2 h at room temperature to avoid unspecific staining. The sections were then incubated with both rabbit primary antibody for anti-GFAP (1 : 200, Abcam) or anti-DCX (1 : 200, Abcam) and mouse monoclonal primary antibody anti-BrdU (1 : 200, Abcam). Briefly, sections were incubated with both primary antibodies overnight at 4°C, followed by anti-rabbit Alexa-488 IgG and anti-mouse Alexa-594 IgG (Molecular probes, OR, USA) for 1 h at room temperature. The stained sections were captured with Axio Observer Z1 and a DE/LSM700 confocal microscope (Carl ZEISS). The numbers of BrdU-positive cells, BrdU/DCX-positive cells, BrdU/GFAP-positive cells in the SVZ, and GFAP-positive cells in the striatum were counted manually on each capture. To minimize the possibility of observer bias, an independent observer that did not know the expected results manually counted the cells bilaterally in three continuous striatal sections, and the cell counts were confirmed three times.

### 2.6. Statistical Analysis

All data are shown as the mean ± standard error of the mean (SEM). The Prism 5 for Windows program (GraphPad Software Inc., La Jolla, CA, USA) was used for statistical analysis. All data were analyzed by one-way analysis of variance (ANOVA) using the Scheffe's post hoc test.* P* values < 0.05 were considered statistically significant.

## 3. Results

### 3.1. Neuroprotective Effect of Acupuncture against MPTP Toxicity

**Figure 2:**
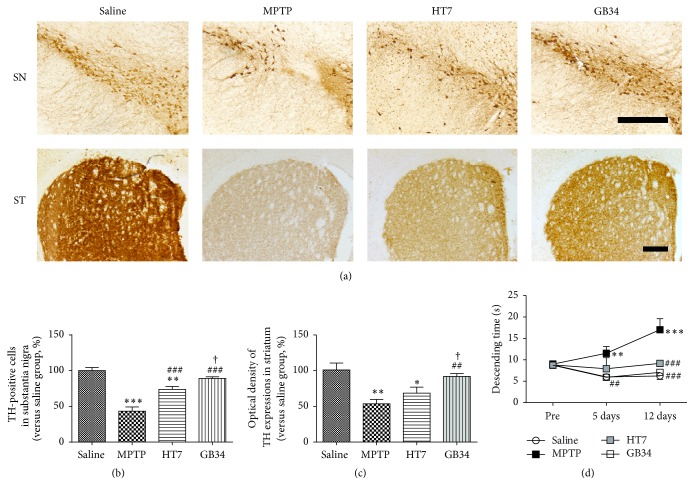
Effect of acupuncture stimulation on 1-methyl-4-phenyl-1,2,3,6-tetrahydropyridine- (MPTP-) induced dopaminergic neuronal death and behavioral dysfunction. (a) Tyrosine hydroxylase- (TH-) specific immunohistochemical staining in the substantia nigra (SN) and striatum (ST). (b) The number of TH-positive cells in the SN. (c) The optical density of TH-positive neurons in the ST. (d) The result of the pole test. Acupuncture stimulation at GB34 suppresses MPTP-induced dopaminergic neuronal death in the SN and the ST and alleviates the MPTP-induced behavioral dysfunctions. Scale bar = 200 *µ*m. Data are shown as the mean ± standard error of the mean. ^*∗*^*P* < 0.05, ^*∗∗*^*P* < 0.01, and ^*∗∗∗*^*P* < 0.001 versus saline group, ^##^*P* < 0.01 and ^###^*P* < 0.001 versus the MPTP group, and ^†^*P* < 0.05 versus HT7 group.

The number of TH-positive cells in the SN of the MPTP group (43.32 ± 5.85%) was significantly reduced relative to that of the saline group (100.00 ± 4.55%,* P* < 0.001). The number of TH-positive cells in the HT7 group (73.79 ± 4.13%) was significantly decreased relative to that of the saline group (*P* < 0.01), but the number was significantly higher than that of the MPTP group (*P* < 0.001). The number of TH-positive cells in the GB34 group (89.05 ± 2.44%) was significantly increased relative to that of the MPTP group (*P* < 0.001) and that of the HT7 group (*P* < 0.05), while it was not significantly different from that of the saline group (Figures 2(a) and 2(b)).

When compared to TH immunoreactivity in the striatum of the saline group (100.00 ± 9.43%), those of the MPTP group (53.64 ± 6.05%,* P* < 0.01) and the HT7 group (68.55 ± 8.33%,* P* < 0.05) were significantly reduced. TH immunoreactivity of the GB34 group (92.00 ± 3.93%) was significantly increased relative to those of the MPTP group (*P* < 0.01) and the HT7 group (*P* < 0.05), but not significantly different from that of the saline group (Figures 2(a) and 2(c)).

### 3.2. Effect of Acupuncture on MPTP-Induced Behavioral Change

Before MPTP administration, descending times of mice in all groups were not significantly different. Five days after the first MPTP administration, the descending time of the MPTP group (11.53 ± 1.58 s) was significantly delayed relative to that of the saline group (6.00 ± 0.23 s,* P* < 0.001); however, that of the GB34 group (5.92 ± 0.27 s) was significantly lower than that of the MPTP group (*P* < 0.01) and not significantly different from that of the saline group. Twelve days after the first MPTP administration, the descending time of the MPTP group (17.03 ± 2.56 s) was significantly delayed relative to that of the saline group (6.24 ± 0.82 s,* P* < 0.001); however, those of the GB34 (7.08 ± 0.52 s) and the HT7 (9.17 ± 0.59 s) groups were significantly lower than that of the MPTP (*P* < 0.001 for each group) group and not significantly different from that in the saline group (Figure 2(d)).

### 3.3. Effect of Acupuncture on Cell Proliferation in SVZ

**Figure 3:**
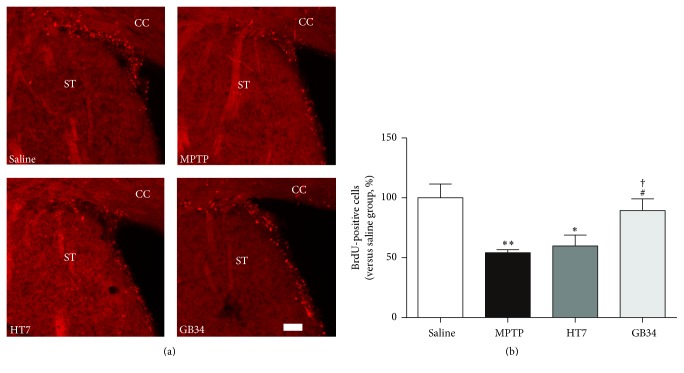
Effect of acupuncture stimulation on MPTP-induced reduction of cell proliferation in the subventricular zone (SVZ). (a) BrdU-specific immunofluorescence staining in the SVZ. (b) The number of BrdU-positive cells in the SVZ. MPTP reduces the number of BrdU-positive cells in the SVZ, but the acupuncture stimulation at GB34 restored it significantly. CC: cerebral cortex. ST: striatum. Scale bar = 20 *µ*m. Data are shown as the mean ± standard error of the mean. ^*∗*^*P* < 0.05 and ^*∗∗*^*P* < 0.01 versus saline group, ^#^*P* < 0.05 versus MPTP group, and ^†^*P* < 0.05 versus HT7 group.

When compared to the number of BrdU-positive cells in the SVZ of the saline group (100.00 ± 11.55%), the numbers in the MPTP (54.25 ± 2.48%,* P* < 0.01) and the HT7 (59.86 ± 9.13%,* P* < 0.05) groups were significantly reduced. However, the level of these cells in the GB34 group (89.05 ± 2.44%) was significantly increased relative to the MPTP and HT7 (*P* < 0.05 for each group) groups, while it was not significantly different from that of the saline group (Figure 3).

### 3.4. Number of BrdU/DCX-Positive Cells in SVZ

**Figure 4:**
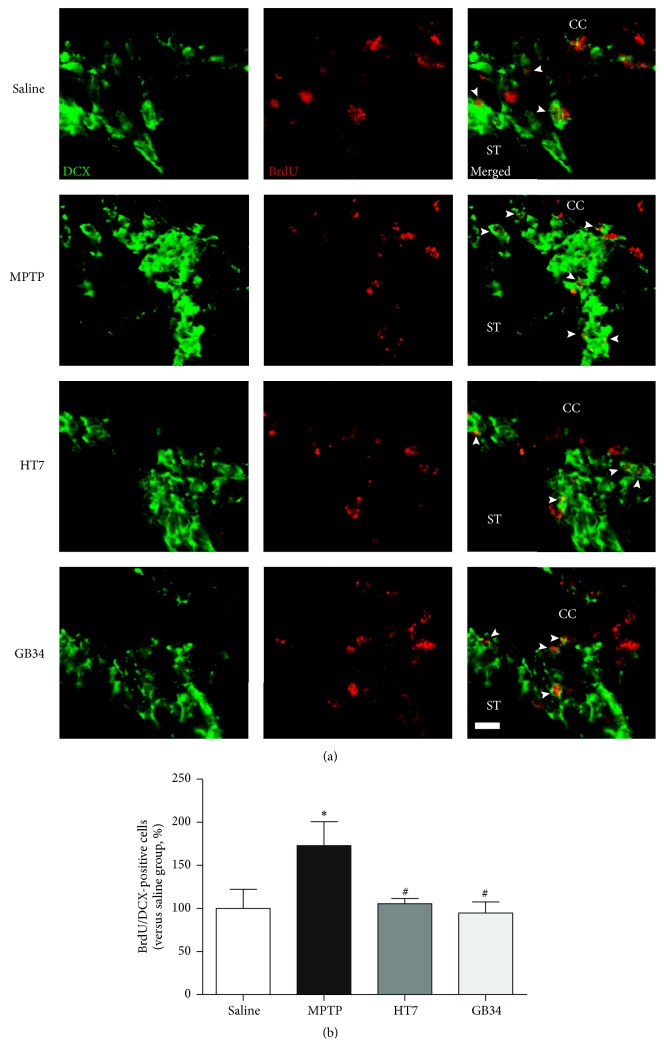
Histological images and graphs of BrdU and doublecortin- (DCX-) positive cells in the SVZ. (a) BrdU and doublecortin- (DCX-) specific immunofluorescence staining. (b) The number of BrdU/DCX-positive cells in the SVZ. The number of the BrdU/DCX-double stained cells increased after MPTP administration, but acupuncture stimulation suppressed this increase. CC: cerebral cortex. ST: striatum. Scale bar = 10 *µ*m. Data are shown as the mean ± standard error of the mean. ^*∗*^*P* < 0.05 versus saline group and ^#^*P* < 0.05 versus MPTP group.

The number of BrdU/DCX-double stained cells in the SVZ of the MPTP group (172.94 ± 27.71%) was significantly increased relative to that of the saline group (100.00 ± 22.29%,* P* < 0.05). However, the level of these cells in the HT7 (105.55 ± 5.31%) and GB34 (94.62 ± 12.94%) groups was significantly decreased relative to that of the MPTP group (*P* < 0.05 at each group) and not significantly different from that of the saline group (Figure 4).

### 3.5. Number of BrdU/GFAP-Positive Cells in SVZ

**Figure 5:**
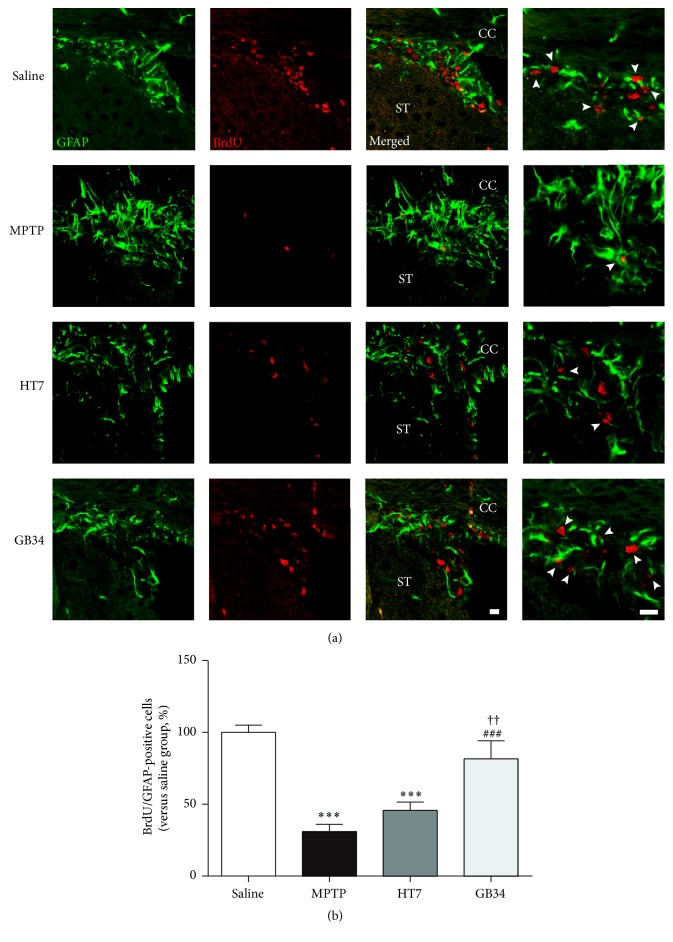
Histological images and graphs of BrdU and glial fibrillary acidic protein- (GFAP-) positive cells in the SVZ. (a) BrdU and glial fibrillary acidic protein- (GFAP-) specific immunofluorescence staining. (b) The number of BrdU/GFAP-positive cells in the SVZ. Based on the results of BrdU (red) and GFAP (green)-positive cells in the SVZ, the number of the BrdU/GFAP-double stained cells in the SVZ decreased after MPTP administration, but acupuncture stimulation suppressed this decreased. CC: cerebral cortex. ST: striatum. Scale bar = 10 *µ*m. Data are shown as the mean ± standard error of the mean. ^*∗∗∗*^*P* < 0.001 versus saline group, ^###^*P* < 0.001 versus MPTP group, and ^††^*P* < 0.01 versus HT7 group.

When compared to the number of BrdU/GFAP-double stained cells in the SVZ of the saline group (100.00 ± 5.06%), the levels in the MPTP (30.92 ± 5.09%) and the HT7 (45.74 ± 5.87%) groups were significantly reduced (*P* < 0.001 at each group). However the level of the GB34 group (81.62 ± 12.60%) was significantly increased relative to those of the MPTP (*P* < 0.001) and the HT7 (*P* < 0.01) groups and not significantly different from that of the saline group (Figure 5).

### 3.6. Number of GFAP-Positive Cells in Striatum

**Figure 6:**
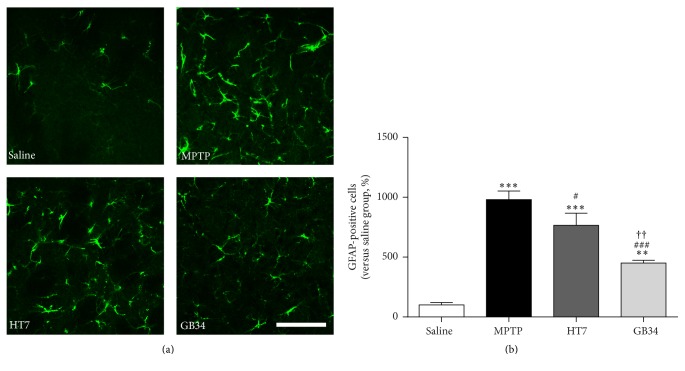
GFAP-positive cells-specific immunofluorescence staining in the striatum. (a) Representative images of GFAP-positive cells. (b) The number of GFAP-positive cells in the striatum. MPTP increases the number of GFAP-positive cells in the striatum, but acupuncture stimulation at GB34 restored it significantly. Scale bar = 100 *µ*m. Data are shown as the mean ± standard error of the mean. ^*∗∗*^*P* < 0.01 and ^*∗∗∗*^*P* < 0.001 versus saline group, ^#^*P* < 0.05 and ^###^*P* < 0.001 versus MPTP group, and ^††^*P* < 0.01 versus HT7 group.

When compared to the number of GFAP-positive cells in the striatum of the saline group (100.00 ± 20.87%), the levels of the MPTP (980.74 ± 71.36%,* P* < 0.001), HT7 (765.00 ± 102.48%,* P* < 0.001), and GB34 (450.00 ± 23.33%,* P* < 0.01) groups were significantly increased. However, the levels of these cells in the HT7 and the GB34 groups were significantly decreased relative to that in the MPTP group (*P* < 0.05 and* P* < 0.001, respectively) and that in GB34 group was significantly decreased relative to that in the HT7 group (*P* < 0.01, Figure 6).

## 4. Discussion

Chronic or acute intraperitoneal administration of MPTP is widely used to model the DA deficit of PD. MPTP crosses the blood-brain barrier, oxidizes to 1-methyl-4-phenylpyridinium, is taken up by DA neurons, and impairs mitochondrial function by inhibiting complex I. Moreover, reduced cell proliferation was observed in the SVZ of MPTP-injected mice [15]. In this study, MPTP administration caused DA neuronal death in the SN and the striatum and suppressed cell proliferation in the SVZ of the mice, similar to previous studies. Interestingly, acupuncture stimulation at GB34 suppressed the MPTP-induced DA neuronal death and restored the MPTP-induced impaired cell proliferation. Acupuncture stimulation at HT7 also suppressed behavioral dysfunction and DA neuronal death in the SN, but the effects were not as strong as those of acupuncture stimulation at GB34 and did not alleviate the impaired cell proliferation.

Degeneration of the nigrostriatal pathway causes striatal dopamine deficiency, which leads to symptoms of PD. Among the nigrostriatal pathways, the motor symptoms of PD are mainly due to DA neuronal degeneration in the SN, and PD-induced cognitive impairments are related to the neuronal death in the striatum [19, 20]. In this study, both acupuncture stimulations at GB34 and HT7 significantly reduced DA neuronal death in the SN against MPTP toxicity and suppressed MPTP-induced behavioral dysfunction, which indicates that the alleviation of the behavioral impairment with the acupuncture stimulations was due to suppressing DA neuronal death in the SN.

Neurogenesis is the process by which neurons are generated from neural stem cells and progenitor cells, which can be demonstrated by BrdU. In the present study, we found that acupuncture stimulation at GB34 restored the reduction of BrdU-positive cells in the SVZ by MPTP administration, confirming that the acupuncture stimulation can restore MPTP-induced neurogenesis impairment.

Although acupuncture stimulation at GB34 restores neurogenesis impairment in the SVZ of MPTP-administrated mice, it is still not clear which cells are divided into DCX-positive neural progenitor cells (A cells) or GFAP-positive neural stem cells (B cells). Therefore, we investigated the numbers of BrdU/DCX-double stained and BrdU/GFAP-double stained cells in the SVZ. The results revealed that MPTP administration caused an increase of BrdU/DCX-positive cells and a reduction of BrdU/GFAP-positive cells in the SVZ. The increase of BrdU/DCX-positive cells can be explained by a self-repair function in which newly proliferated cells in the SVZ were differentiated to neurons to compensate for MPTP-induced neuronal death [21, 22]. Similar trends have also been observed in animal models of other brain diseases such as stroke and Huntington's disease [23, 24]. Therefore, the increase of BrdU/DCX-positive cells in the SVZ suggests that new neurons were provided against MPTP-induced neuronal death.

Astrocytes can protect neurons by scavenging radicals and glutamate; however they destruct neurons under conditions of chronic inflammatory stress including MPTP administration [25]. In this study, the number of BrdU/GFAP-positive cells in the SVZ was reduced after MPTP injection, whereas that of GFAP-positive cells in the striatum was increased. There results indicate that the reduction of BrdU/GFAP-positive cells may be an extension of that of BrdU-positive cells in SVZ and have nothing to do with the increase of GFAP-positive cells in the striatum. Taken together, these findings indicate that MPTP administration suppresses cell proliferation in the SVZ, although it increases the number of BrdU/DCX-double stained cells to compensate for neuronal death.

In this study, acupuncture stimulation at GB34 normalized the MPTP-induced increase of the BrdU/DCX-positive cells and decrease of the BrdU/GFAP-positive cells, which could be explained from two aspects. Specifically, acupuncture stimulation directly affected the neurogenesis in the SVZ. Additionally, it suppressed the MPTP-induced DA neuronal death, so neurogenesis in the SVZ was maintained at a normal level and the compensation mechanism was not activated. But the hypotheses could not be resolved by the data in this study; therefore more additional studies are warranted.

In case of acupuncture stimulation at HT7, it did not alleviate the impaired cell proliferation although it suppressed behavioral dysfunction and DA neuronal death in the SN, which suggests that the neuroprotective mechanism is not related to neurogenesis. To clarify it, more rigorous studies are needed.

In conclusion, acupuncture stimulation at GB34 alleviated MPTP-induced motor dysfunction, protected against MPTP-induced DA neuronal death in the SN and the striatum, and restored MPTP-induced neurogenesis impairment. Overall, these results indicate that acupuncture stimulation can be an alternative therapy for PD.
